# Dr. Storer on Some Memoirs of Dr. Milward

**Published:** 1812-01

**Authors:** Edward Storer


*To the Editors of the Medical and Physical Journal.*

GENTLEMEN,

YOUR last most respectable number contains an inquiry from the ingenious Dr. Henderson, of Golden-square, respecting the progress made by the late Dr. Edward Milward, in his "circular invitatory letter to all orders of learned men, concerning an attempt, or essay, towards an history of the lives, deaths, writings, characters, and opinions, of the most celebrated British Physical and Chirurgical authors, &c." Being in possession of some valuable memoirs of the above eminent physician (Dr. Milward,) and from the authority of these memoirs, the Doctor's scheme was forgotten and unfinished. He found the undertaking and the objects objects too multifarious to be accomplished in any satisfactory manner by the labours of an individual, they were therefore wholly abandoned.

It may probably be useless in me to mention, that the well-known Dr. Aikin published, in the year 1780, "Biographical Memoirs of Medicine in Great Britain, from the Revival of Literature to the Time of Harvey;" a work deservedly well received. In the year 1799, a work was published by Mr. Hutchinson, entitled "Biographia Medica, or Historical and Critical Memoirs of the Lives and Writings of the most eminent Medical Characters, that have existed from the earliest Account of Time to the present Period, with a Catalogue of their Literary Productions; in two volumes, octavo." This work has been the vehicle of much and extended information to me, in pursuits of a somewhat similar nature, and in a voluminous inquiry into Medical Biography, in which I am at this moment engaged; my obligations to the author of the last mentioned work will be acknowledged with a proper sense of gratitude and respect. In the mean time, should Dr. Henderson require any further information respecting Dr. Milward, the memoirs in my possession will be very much at his service.

I am, Gentlemen,

Your obedient servant,

EDWARD STORER, M.D.

*December, 8th,* 1811.

